# Identification of conditionally essential genes for *Streptococcus suis* infection in pigs

**DOI:** 10.1080/21505594.2020.1764173

**Published:** 2020-05-18

**Authors:** Jesús Arenas, Aldert Zomer, Jose Harders-Westerveen, Hester J. Bootsma, Marien I. De Jonge, Norbert Stockhofe-Zurwieden, Hilde E. Smith, Astrid De Greeff

**Affiliations:** a Department of Infection Biology, Wageningen Bioveterinary Research (WBVR), Lelystad, The Netherlands; b Unit of Microbiology and Immunology, Faculty of Veterinary, University of Zaragoza, Zaragoza, Spain; c Department of Infectious Diseases and Immunology, Faculty of Veterinary Medicine, Utrecht University, Utrecht, The Netherlands; d Section Pediatric Infectious Diseases, Laboratory of Medical Immunology, Radboud, Institute for Molecular Life Sciences, Radboudumc, Nijmegen, The Netherlands

**Keywords:** *Streptococcus suis*, pathogenesis, zoonotic pathogen, infection, transposon mutagenesis, Tn-Seq

## Abstract

Streptococcus suis

is a Gram-positive bacterium and zoonotic pathogen that causes meningitis and sepsis in pigs and humans. The aim of this study was to identify genes required for *S. suis* infection. We created Tn-Seq libraries in a virulent *S. suis* strain 10, which was used to inoculate pigs in an intrathecal experimental infection. Comparative analysis of the relative abundance of mutants recovered from different sites of infection (blood, cerebrospinal fluid, and meninges of the brain) identified 361 conditionally essential genes, i.e. required for infection, which is about 18% of the genome. The conditionally essential genes were primarily involved in metabolic and transport processes, regulation, ribosomal structure and biogenesis, transcription, and cell wall membrane and envelope biogenesis, stress defenses, and immune evasion. Directed mutants were created in a set of 10 genes of different genetic ontologies and their role was determined in *ex vivo* models. Mutants showed different levels of sensitivity to survival in whole blood, serum, cerebrospinal fluid, thermic shock, and stress conditions, as compared to the wild type. Additionally, the role of three selected mutants was validated in co-infection experiments in which pigs were infected with both wild type and isogenic mutant strains. The genetic determinants of infection identified in this work contribute to novel insights in *S. suis* pathogenesis and could serve as targets for novel vaccines or antimicrobial drugs.

## Introduction

*Streptococcus suis* is the major etiological agent of streptococcal disease in pigs, a systemic infection that is the major cause of morbidity and mortality in young pigs leading to significant economic losses to the swine industry worldwide. The bacterium resides asymptomatically in the oropharynx, gut, and genitals of pigs as part of the microbiota. Particularly during the nursery period in piglets of 4 to 10 weeks old, *S. suis* can cross dermal and mucosal barriers and reach the bloodstream causing arthritis, sepsis, meningitis, and other pathologies [1,2]. *S. suis* can also infect humans and is one of the most common causes of meningitis in pork-consuming countries in Asia [3]. The rapid onset of disease together with the emergence of multidrug-resistant strains makes adequate treatment and control difficult.

Vaccination is used extensively to prevent *S. suis* infections. Current immunization strategies focus on commercial and autologous whole-cell bacterins. These vaccine formulations only induce protection against the serotype present in the bacterin and are not equally efficacious for all serotypes [4]. Alternatives are live attenuated or subunit-based vaccines. Several live-attenuated vaccine strategies were described; including those based on auxotrophic mutants for aromatic amino acids [5], or mutants lacking virulence-associated factors [6,7]. In general, these vaccine preparations show a better efficacy than bacterins; however, there are sometimes concerns regarding safety when the mutant is not fully attenuated [8,9]. Interestingly, a low virulent strain XS05 and a double mutant in two surface-exposed proteins (SspepO and SspspC) were recently used as live vaccines [10,11], and induced protection against challenge experiments with strains of different serotypes, indicating that these are promising vaccine candidates. In contrast to live-attenuated vaccines, subunit-based vaccines are safe; however, costs are the main drawback for their further development. In addition, the high diversity of cell surface structures in *S. suis* makes it difficult to identify protective antigens common to all, while the identification of one or a combination of universal antigens is required to develop a broad protective formulation. Although several promising subunit vaccine candidates have been described, there is not yet a commercial cross-protecting vaccine on the market.

Conditionally essential genes required for *S. suis* infection represent attractive targets for the design of subunit or live-attenuated vaccines and other innovative interventions. The main discovery approach for novel antigens has been labor intensive focusing on a few genes. A non-biased and systematic selective method requires the creation of large libraries of mutants combined with genome-wide screens using hybridization [12], footprinting [13], or next-generation sequencing (Tn-Seq) [14]. In Tn-Seq, the gene function of each mutated gene is disrupted by random insertion of a transposon into the gene. The resulting Tn-Seq library is subjected to selective conditions where the relative abundance of every mutant in the pooled population is compared between the pooled input library and the output population after exposure to the selective condition. Variation in the abundance of the mutants between input and output, e.g. after different growth conditions, indicates the relevance of genetic regions for the fitness of bacteria under the selective condition. Tn-Seq has been successfully applied to identify sets of conditionally essential genes in more than 40 microorganisms, including diverse Streptococcus species [14–16], an important evidence base that this approach could also be applied to *S. suis*.

The goal of this work was to identify genes of *S. suis* that are conditionally essential during infection of pigs using Tn-Seq. A large number of putative essential genes were identified in different sites of infection and the essential role of a subset of genes during the infection was confirmed with directed mutants using a panel of *in vivo* mimicking conditions. Finally, we confirmed the essential role of some of these mutants in the *in vivo* infection process during an *in vivo* experimental infection.

## Methods

### Strains and culture conditions

*S. suis* strain 10 was previously described [17]. Bacteria were grown at 37°C and 5% of CO_2_ on Columbia blood base agar plates (Oxoid) containing horse blood 6% (vol/vol) and supplemented with 100 µg/ml of spectinomycin when appropriate for a selection of transformants. For bacterial liquid cultures, one colony collected from solid culture was propagated in Todd-Hewitt Broth (THB, Oxoid) and incubated overnight in 100 ml-culture bottles under the same conditions.

For growth under *in vivo* mimicking conditions, overnight cultures were diluted in fresh THB supplemented with 0.5 mM 2,2-Bipyridyl (Bipyridyl), 10 mM sodium fluoride or 3 mM H_2_O_2_, incubated in a Bioscreen C instrument (Thermo Scientific) at 34°C, 37°C, or 42°C and the optical density at 600 nm was monitored every 20 min. For studying the growth of the bacteria in different host fluids, overnight THB cultures were diluted to around 10^6^ CFU/ml in Dulbecco’s phosphate-buffered saline, and bacteria were inoculated in fresh porcine blood, 100% commercial porcine serum (Sigma-Aldrich) or porcine cerebrospinal fluid (CSF) and incubated at 37°C and 5% of CO_2_. Blood and CSF were collected from healthy 3–6 -week-old specified pathogen-free piglets from a high health farm, as piglets are most at risk for streptococcal suis infection at 4–10 weeks. Bacterial survival was monitored by CFU counting using solid agar plates.

### *Preparation of* S. suis *Tn-Seq libraries*

*S. suis* strain 10 mariner transposon mutant libraries were prepared as described previously for *S. pneumoniae* [18], except for the procedure of *S. suis* transformation [19]. Briefly, 0.52–1 µg/ml of purified genomic DNA of *S. suis* strain 10 was incubated with a similar amount of Himar I mariner transposon in the presence of purified transposase at 30°C for 4 h. Mutagenized genomic DNA was repaired with T4 DNA polymerase and *E. coli* DNA ligase. 10 µl of the ligation mixture (approximately 1 µg/ml) was used for transformation of 100 µl of *S. suis* strain 10 using the previously described peptide-induced competence system [19], plated on THB-agar plates with spectinomycin (100 µg/ml) and incubated for 16 h in 37°C under 5% CO_2_. The colonies were gently removed from the plate by adding 2 ml of THB and scraping gently with a spatula. The bacterial suspension was grown in THB containing spectinomycin (100 µg/ml) for 2 h in 37°C under 5% CO_2_ and when required stored at −80°C in THB containing 15% glycerol.

### Animal experiments

#### Ethical statement

The animal experiments described below were approved by the Central Authority for Scientific Procedures on Animals according to the Dutch law on animal experimentation in accordance with current legislation and fostering the principles of the 3 Rs of animal testing: replacement, reduction, and refinement (permit numbers AVD401002015140, 2014079, 2016077).

#### Porcine meningitis model

In a pilot experiment to develop a porcine meningitis model, we used four piglets of 6 weeks old (TOPIGS breed, originating from a high health status farm). Piglets were allowed to acclimatize for a week and were fastened for 16 h with ad libitum access to water before the surgical procedure. The surgical procedure aimed to directly inoculate the subarachnoid space by intrathecal cannulation of the cisterna magna of the cervical column, based on the procedure previously described by Romagnoli et al. [20]. In short, piglets were anesthetized using an intramuscular administration of Ketamine (15 mg/kg body weight Ketalar) and Midazolam (0.75 mg/kg body weight Dormicum). Intravenous catheters were inserted in both ear veins and used for continuous administration of 2.5% Glucose/0.45% Sodium Chloride and for the continuation of intravenous anesthesia by the administration of fentanyl (Sufenta®, Janssen; 10 µg/kg/h) and 6.5–30 mg of Propofol (Propovet). Animals were positioned in sternal recumbency without any flexion of the head and the puncture site of the cisterna magna was marked. A 70 mm 22-gauge needle with an i.v. catheter (Vasofix®, Braun Meslsungen, DE_catheter) was used for puncture and the correct intrathecal location was confirmed by a collection of 1 ml CSF. The animals were infected intrathecally and intravenously with *S. suis* strain 10. Two factors were important to determine the bacterial dose: *i*) at least 10^2^ CFUs of all clones of the library should be present in the inoculum, and *ii*), sufficient amount of each surviving clone should be re-isolated after infection (at minimum 10^2^ CFUs). Therefore, we used 8.7 × 10^8^ CFUs as inoculation dose. Rectal body temperature of piglets was monitored hourly; blood and CSF samples were also collected every h to determine bacterial load as a function of time by plating on Colombia agar and CFU counting after overnight incubation. After 12 h of infection, the piglets were euthanized using Euthasol® (AST Beheer BV, Oudewater, The Netherlands) and subjected to necropsy. During necropsy, the brain surfaces were washed with PBS to collect adherent bacteria. Organ specimens of infected animals were weighed, mixed with one volume of THB medium and homogenized in a Stomacher 400 (Seward). The presence of bacteria in homogenized organs (spleen, kidney, liver, peritoneum, thorax, heart, lung, cerebrum, and cerebellum) was determined by plating and CFU determination. Tissue samples of cerebrum, cerebellum, and spinal cord were collected, fixed in 4% of formaldehyde, and embedded in paraffin. Tissue slides from the brainstem, the cerebellum, and the cerebrum were stained with hematoxylin and eosin to determine the presence of inflammatory cells and the degree of inflammation.

Based on the results of the pilot experiment, genes conditionally essential during *in vivo* infection were selected using the Tn-Seq strategy. To do this, seven 8-week-old piglets were infected directly intrathecal by cannulation of the cisterna magna of the cervical column as described above, with 8.7 × 10^8^ CFU of *S. suis* Tn-Seq libraries in strain 10. The results of the pilot study indicated that after 5 h the number of recovered bacteria reached a maximum. Therefore, after 5 h of infection, pigs were anesthetized and euthanized as described above.

#### Co-infection experiments

Fifteen 7-week-old piglets, crossbreeds of Great Yorkshire and Dutch Landrace, were obtained from sows by cesarean sections and raised without maternal antibodies from colostrum (Cesarean Derived Colostrum Deprived piglets), and raised under stringent hygienic conditions. Piglets were used in co-infection experiments to determine the virulence of mutants in a competitive model. At the start of the experiment tonsil swabs of piglets were screened for the absence of *S. suis* serotype 2 isolates. After an acclimatization period of 3 days, piglets were infected intravenously with 10^6^ CFU of strain 10 and co-infected with the same amount of one of the isogenic mutants as previously described [21]. After infection, blood was extracted at a daily base to allow white blood cell counting, and piglets were observed for clinical symptoms and behavior three times a day at 8 h intervals. The rectal body temperature was determined three times a day. As soon as animals reached the predefined humane endpoints, the piglets were euthanized and subjected to necropsy. During necropsy, organs, as well as joints and CNS, were examined for gross pathology and histology. Blood samples were collected and organs were extracted and homogenized as described above. The ratios of the mutants over wild-type bacteria in blood and organs were determined by plating on Colombia agar media with and without spectinomycin. Growth on plates with spectinomycin represents the amount of mutant isolate, whereas growth on the plates without antibiotics represents the combined number of wild-type bacteria and mutant derivative.

### DNA isolation

Bacterial DNA was extracted from bacterial isolated from the meninges, the CSF, and the blood of infected piglets. Two methods were followed, either direct DNA isolation or DNA isolation after enrichment of the bacteria. To enrich for bacteria, samples were inoculated in warm THB medium and allowed to grow for 1 h at 37°C. After enrichment, the bacteria were collected by centrifugation and washed three times with PBS. Pellets were used for DNA isolation. For the direct DNA isolation, bacteria were collected from samples by centrifugation and washed three times with PBS. DNA was isolated using the genomic-tip 20/G columns (Qiagen, Hilden, Germany) following the manufacturer’s instructions including the pretreatment for Gram-positive bacteria. DNA quantity was determined using a Nanodrop (Thermo Fischer, Wilmington, USA). To quantify the amount of prokaryotic and eukaryotic DNA, a qPCR was performed using primers for the capsule gene (*cps2 J*) of *S. suis* and primers for the porcine actin gene (*actB*) (PrimerDesign, Chandler’s Ford, UK). PCR reactions were performed using SYBR Green Master Mix (Thermo Fischer) according to the manufacturer’s instructions. PCR reactions were run in a 7500 real-time PCR machine (Applied Biosystems). Based on the ratio between prokaryotic and eukaryotic DNA, it was decided that growth in THB was required for samples obtained from blood to obtain sufficient DNA. The resulting DNA was used for sequencing.

### Tn-Seq sequencing and data analysis

Tn-Seq libraries were sequenced before and after infection as described previously [22] using Illumina Hi-seq in triplicate or quadruplicate resulting in a total of 27 Tn-Seq datasets. All sequencing data are available at the Sequence Read Archive under project accession PRJEB35559. Tn-Seq data analysis was performed as described previously [23]. Briefly, FASTQ files of the Tn-Seq results were imported to the web-based interface ESSENTIALS (bamics2.cmbi.ru.nl/websoftware/essentials) and extracted with default settings with *S. suis* P1/7 as the reference genome. Count data (i.e. pseudoreads) were generated per unique sequence read or per gene and corrected by locally weighted scatterplot smoothing (LOESS) for the bias in Tn-Seq data caused by the increase in available DNA close to the origin of replication (ORI). Normalization factors were calculated using the trimmed mean of M values (TMM). Pseudoreads in the control and target samples were tested for significant differences by using the negative binomial model in EdgeR implemented in ESSENTIALS [24]. The dispersion was estimated with the Cox-Reid profile adjusted likelihood method to adjust for the library as co-factor and the variance was modeled using tagwise dispersion. The prior.n value to determine the amount of smoothing of tagwise dispersions was set at 5. P value adjustment (adjusted P ≤ 0.05) was based on the Benjamini-Hochberg procedure. Genes were considered essential for a condition when they had a log2 fold difference between control and target condition with a Benjamini-Hochberg adjusted p-value <0.05. Insertions in essential genes for in vitro cultures were underrepresented and they were ruled out from the study. We also excluded putative essential genes sustaining no insertions, duplicated genes, disrupted and pseudogenes, small genes, intergenic regions, and no coding genes. For the wide representation of Tn-Seq libraries, mutants that have summed reads of 25 were considered and generated with Circos [25]. The final gene list was analyzed with Kyoto Encyclopedia of Genes and Genomes orthology [26] and the SEED databases [27]. Gene ontology analysis was performed to determinate the functional enrichment.

### Generation of directed mutants

To obtain knockout mutants in *S. suis* strain 10 (SSU0114, *glA*, SSU0469, *fhs*, SSU0883, *penA*, SSU1501, SSU1869, SSU1940), DNA fragments corresponding to flanking regions of the genes of interest and a spectinomycin-resistance cassette were amplified from chromosomal DNA of *S. suis* strain 10 using primers described in Table S1. A High Fidelity Polymerase kit (Roche Diagnostics GmbH) was used for the preparation of PCR reactions. PCR products were purified by column purification (Qiagen), analyzed on 1% agarose gels and purified using commercial kits (Qiagen). DNA segments of flanking genes and that of a spectinomycin-resistance cassette were used as a template for a new PCR reaction using primers flanking the extreme segments (Table S1). For the creation of the mutant 10ΔSSU1869, a DNA fragment containing the spectinomycin-resistance cassette (replacing the *troA* gene) and both flanking regions was amplified from the serotype 9 mutant strain 8067Δ*troA* [28] (equivalent to SSU1869) with primers described in Table S1. The final PCR products were used to transform *S. suis* strain 10 as described [19]. PCR assays and sequencing were used to confirm the mutations.

### *Statistical analysis to characterize the directed mutants* in vitro *and* in vivo

GraphPad Prism was used for statistical analyses. For comparison of growth curves, the relative area under the curve obtained in three independent experiments performed in triplicate was used, and an unpaired t-test was used for statistical comparisons. P values of <0.05 were considered significant.

## Results and discussion

### Fitness determinants in a novel meningitis infection model

**Figure 1.**
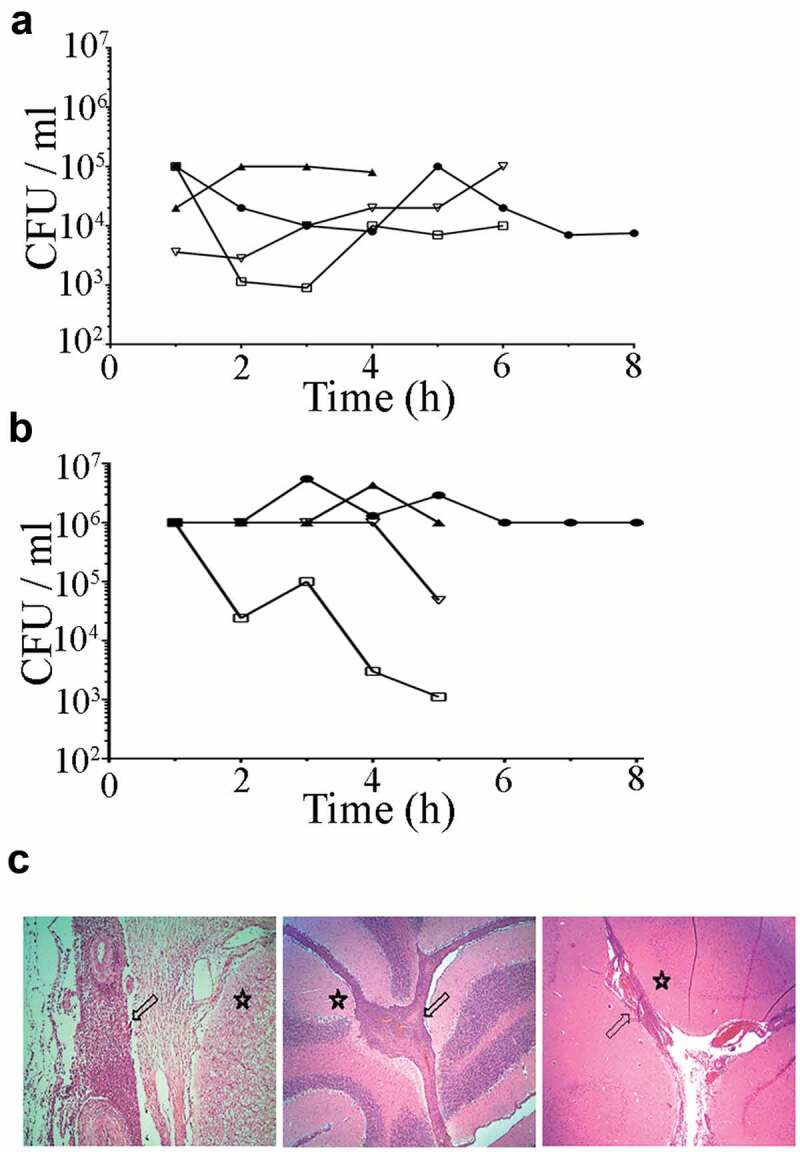
Bacterial loads in (a) blood and (b) CSF after intravenous and intrathecal infection of pigs with *S. suis* strain 10 and (c) histological changes in central nervous tissues. Bacterial loads were calculated by CFU determination each hour post infection (hpi). Each symbol and line represent one individual animal of four. Histological examinations show typical suppurative meningitis (arrows) in various degrees in the spinal cord (left panel), cerebellum (central panel), and cerebrum (right panel) of inoculated pigs. Star symbol indicates the location of nervous tissue.

To screen for essential determinants of *S. suis* infection during meningitis and septicemia, we employed a novel intrathecal infection model thereby inoculating piglets directly into the subarachnoid space with transposon libraries of pathogenic *S. suis* serotype 2 strain 10. *In vivo* selection of Tn-Seq libraries requires isolation of sufficient bacteria from infected sites. In regular experimental infections using the intravenous inoculation route, it is challenging to retrieve sufficient amounts of bacteria, since they are rapidly dispersed through the whole body via the bloodstream. The inoculation route used in this study bypasses several host barriers to prevent the bottlenecks of infection as described [29]. To determine the feasibility of this *in vivo* Tn-Seq approach, a pilot experiment was conducted to compare intravenous inoculation to intrathecal inoculation into the subarachnoid space. The main questions to be answered were: (1) which inoculation route yields sufficient numbers of bacteria to allow Tn-Seq analysis, and (2) are the observed clinical symptoms representative for an *S. suis* infection? The body temperature of all piglets increased rapidly within 2 h post infection (hpi) (Fig. S1). The amounts of bacteria in blood and CSF fluctuated over time, particularly in blood (Figure 1(a)). In blood, bacterial counts remained below 10^5^ CFU ml^−1^ whereas in CSF it reached almost 10^7^ CFU ml^−1^ in two piglets, while in the two remaining piglets the bacterial load oscillated (Figure 1(b)). Since the piglets were kept under anesthesia for the whole experiment, no clinical scoring was possible. To verify that inoculation directly into the subarachnoid space resulted in similar symptoms of meningitis, as observed during infection in the field, cerebrum, cerebellum, and spinal marrow were subjected to histopathological observations. Visual inspection of histological sections revealed severe suppurative meningitis in all pigs (Figure 1(c)), as demonstrated by a strong influx of neutrophils confirming the typical clinical signs observed for *S. suis* meningitis. Based on this pilot experiment, it was decided that intravenous inoculation would probably not yield enough bacteria. Therefore, the selection of Tn-Seq mutants was performed by infecting seven piglets with the Tn-Seq libraries by intrathecal inoculation. Temperatures of piglets increased within 2–3 hours after infection, but less pronounced than in the pilot study (Fig. S2A). After 5 hpi, we finalized the experiment. High concentrations of *S. suis* Tn-Seq mutants were retrieved from CSF ranging from 10^8^–10^11^ CFU ml^−1^ whereas samples of meninges contained on average 10^7^ CFU ml^−1^ (Fig. S2B). Although no direct inoculation in blood was performed, on average 10^5^ CFU ml^−1^ *S. suis* could be retrieved from blood samples (Fig. S2B). All samples were included for Tn-Seq analyses, although the blood samples required additional enrichment to obtain enough material. Histological studies revealed that the inoculation caused an acute fibrino-purulent meningitis in 66% of the piglets (33% severe to very severe, extended meningitis, 33% mild to moderate meningitis) in all parts of the brain. A focal, mild, or moderate encephalitis was present in the cerebellum in 33% of the cases and in 17% in the brain stem or in the cerebrum. One third of the pigs did not develop meningitis or encephalitis despite the fact that high numbers of Tn-Seq mutants were retrieved from all piglets.

**Figure 2.**
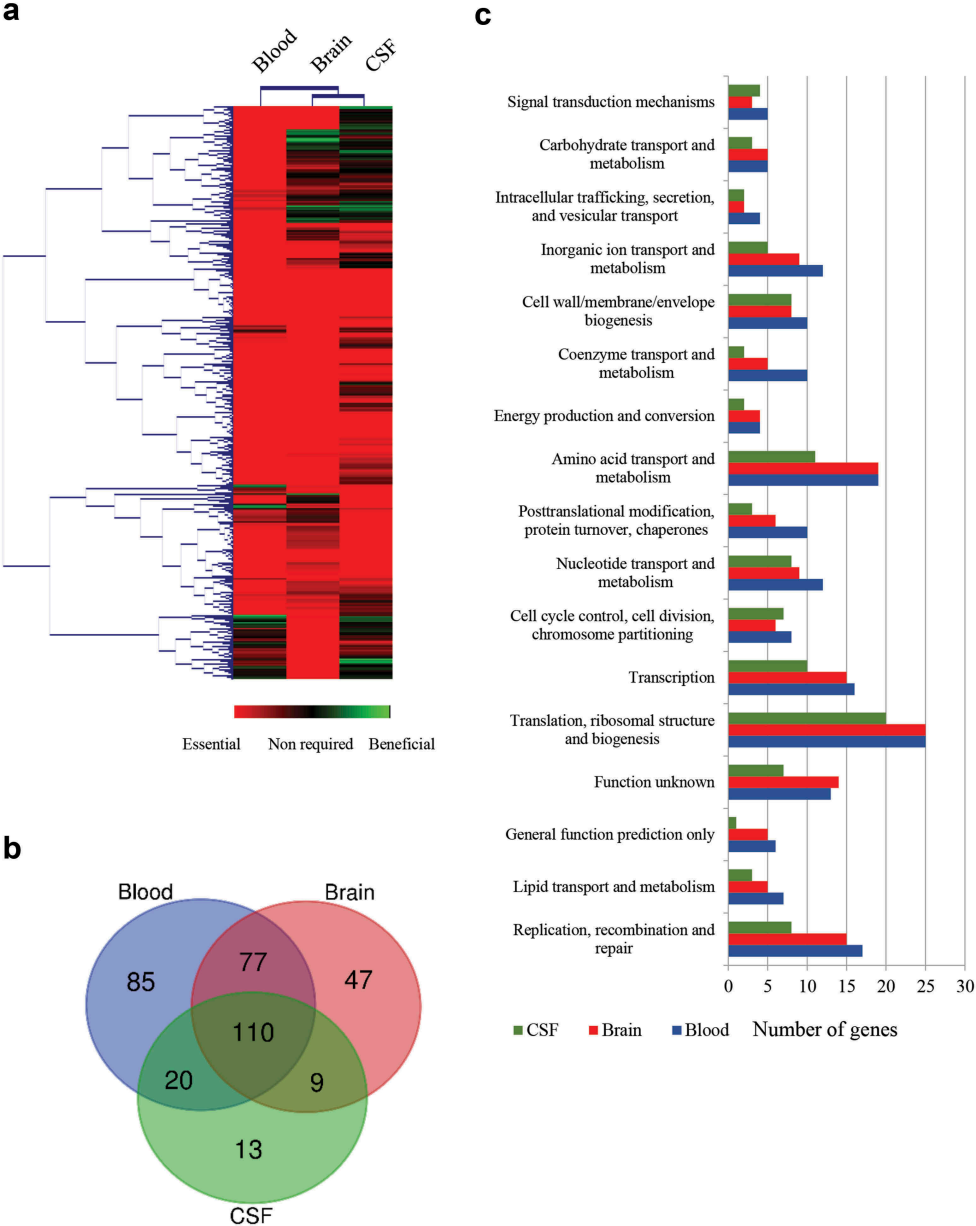
Global overview of Tn-Seq screen. (a) Hierarchical clustering of the complete gene set obtained for each infection site. The level of essentiality for each gene is color-coded as indicated. Genes with an increased log_2_ fold change lower than 2 were considered essential in vivo, higher than 2 were considered as beneficial; otherwise, they were considered as no required. (b) VENN diagram indicating the number of unique or shared essential genes within infection sites. (c) Categorization of gene ontologies of the essential gene set for blood, brain, and CSF according to genome annotations.

Tn-Seq analysis of the input inoculum library revealed 57810 transposon mutations with a higher total number of reads than background (25 reads). These transposon mutants affected a total of 1815 coding and non-coding genes, which covers about 97% of the publicly available P1/7 genome. Fig. S3 shows a map of Tn-Seq mutations in *S. suis* libraries used, including the overall differences between input inoculum and output. DNA sequencing of the output Tn-Seq mutants revealed 32666 unique transposon mutations remaining that correspond to 1635 genes with at least one transposon insertion. Genes were considered as essential when the read counts per gene had at least a two-fold log_2_ decrease and an adjusted *P* value lower than 0.05, as compared with input inoculum. Table S2 in supplemental material lists essential genes for each site of infection along with gene features, fold change differences, and P values. Overall, 361 genes were essential for infection, about 18% of the genome. Figure 2(a) shows a heat-map of all identified genes for each site of infection combined with a hierarchical gene clustering analysis. Two well-defined clusters were identified and gene mutation profiles of CSF and meninges of the brain were closely related and distant of those from blood. Thirty percent of the total of putative essential genes were essential for blood, CSF, and brain, while 40% were uniquely identified in one infection site (Figure 2(b)). According to assigned gene ontology in public genomes, a vast majority of the genes were involved in metabolic and transport processes (33% of annotated genes), translation, ribosomal structure and biogenesis (16% of annotated genes), transcription (9% of annotated genes), replication, recombination and repair (8% of annotated genes), cell wall membrane and envelope biogenesis (6% of annotated genes), among others (Figure 2(c)). Table 1 lists representative genes involved in these processes.

**Table 1.** Essential genes required for growth of *S. suis* in pigs.

			Tn-Seq Fold change^3^		
Locus tag^1^	Gene	Product^2^	Blood	Brain	CSF
*Carbon metabolism*					
SSU0479	*pep*	Phosphoenolpyruvate carboxylase	−8.6	−7.4	−5.0
SSU1636	*pdhB*	Pyruvate dehydrogenase E1 component, Beta sub	−3.4	−2.1	−2.9
SSU1025	*zwf*	Glucose-6-phosphate 1-dehydrogenase	−4	−2.7	−4.3
SSU1839	*tkt*	transketolase	−3.3		−3.7
SSU1315		Lactonase	−9.9		
SSU0936	*deoC*	Deoxyribose-phosphate aldolase	−7.8	−6.9	
SSU1269	*deoB*	Phosphopentomutase		−7.6	
SSU1270	*rpiA*	Ribose-5-phosphate isomerase A	−8.7	−7.6	−4.7
SSU0021	*prsA1*	Ribose-phosphate pyrophosphokinase	−4.3	−2.9	−4.1
SSU0648	*fhs*	Formate-tetrahydrofolate ligase	−5.6	−4.4	−6.1
*Purine and pyrimidine metabolism*					
SSU0122	*rpoB*	DNA-directed RNA polymerase subunit Beta	−5.0	−3.6	−5.1
SSU0123	*rpoC*	DNA-directed RNA polymerase subunit Beta	−4.6	−3.2	−4.7
SSU0816	*guaA*	GMP synthase	−9.8	−8.6	−2.5
SSU0014	*hpt*	Hypoxanthine-guanine Phosphoribosyltransferase	−7.6	−6.5	−4.1
SSU1758	*purA*	Adenylosuccinate synthetase	−3.1	−9.4	−3.6
SSU0037	*purB*	Adenylosuccinate lyase	−4.7	−9.1	−4.0
SSU0378	*gmk*	Guanylate kinase	−3.5		
*Biosynthesis of amino acids*					
SSU0621	*serC*	Phosphoserine aminotransferase	−10.2	−8.9	−3.2
SSU1325		Haloacid dehalogenase	−4.11	−7.8	−5.3
SSU0319		Aminotransferase	−3.1	−5.6	
SSU1720	*cysE*	Serine acetyltransferase	−4.3	−3.0	−4.7
SSU0748	*thrB*	Homoserine kinase	−8.7	−7.6	
SSU0671	*asD*	Aspartate-semialdehyde dehydrogenase	−8.6	−7.4	−3.4
SSU0157	*glnA*	Glutamine synthetase	−4.5		
SSU0262		Threonine synthase	−9.4	−8.3	−3.6
SSU1634	*acoL*	Dihydrolipoamide dehydrogenase	−4.9	−3.6	
*Transporters*					
SSU0114		Metal cation ABC transporter (Permease)	−7.4	−6.3	−8.1
SSU1865		Metal cation ABC transporter membrane protein	−4.2	−2.8	−4.5
SSU0883		Glutamine ABC transporter, glutamine-binding protein/permease protein	−2.2		
SSU1675		Glutamine ABC transporter, glutamine-binding protein/permease protein	−2.3		
SSU1852		Amino acid ABC transporter permease	−3.8	−2.5	−3.7
SSU1662	*oppF*	Oligopeptide transport ATP-binding protein		−5.1	
SSU1583	*manL*	Mannose-specific phosphotransferase system	−7.4	−6.2	
SSU0751	*potA*	Part of permidine/putrescine ABC transporter	−7.8		
SSU0249	*glA*	Putative aquaporin	−9.2		
SSU1869		Metal cation ABC transporter TroA	−5.2	−3.7	−5.5
*Cell envelope biosynthesis*					
SSU0516	*cps2B*	Chain length determinant protein	−7,0	−5.9	−7.7
SSU0519	*cps2E*	Galactosyl transferase	−8.8	−5.6	−4.3
SSU1186	*penA*	Penicillin-binding protein 2b	−8.3	−4.8	−9.4
SSU0018	*merC*	Rod shape-determining protein MreC	−7.8	−6.7	−8.5
SSU0373	*gpsB*	Cell division protein GpsB	−3.4	−2.1	−4.0
SSU1028	*ftsY*	Cell division protein FtsY	−6.2	−5.1	−6.2
SSU1775	*secE*	Preprotein translocase subunit SecE	−3.4	−2.1	−3.7
SSU1615	*secA*	Preprotein translocase subunit SecA	−3.7		
*Membrane and secreted structures*					
SSU1128		Surface anchored protein	−3.9		
SSU0375		Membrane protein	−9.8		
SSU0386		Membrane protein	−7.9	−6.8	−8.6
SSU0473		Membrane protein	−8.6	−7.5	−3.2
*Stress conditions*					
SSU0279	*grpE*	Heat shock protein GrpE	−3.3	−2.0	−3.6
SSU0280	*dnaK*	Molecular chaperone DnaK	−6.2	−4.9	−6.8
SSU0306	*tig*	Trigger factor	−3.8	−7.0	
SSU0023	*recO*	DNA repair protein RecO	−4.2		
*Transcriptional regulators*					
SSU0511		TetR family transcriptional regulator	−5.9		
SSU1326	*ezrA*	Septation ring formation regulator EzrA	−7.7		−4.7
SSU1608		MarR family transcriptional regulator	−4.6	−4.0	
SSU0063	*spxA*	Spx family transcriptional regulator	−7.5	−6.4	
SSU1202	*ccpA*	Catabolite control protein A CcpA	−7.5	−4.5	
SSU0320	*codY*	CodY family transcriptional regulator	−7.5	−6.3	
SSU0869		LysR family transcriptional regulator	−10.1		
*Unknown function*					
SSU0469		Hypothetical protein	−3.6	−2.3	
SSU1501		CsbD-like protein	−5.5		
SSU1940		c-di-AMP phosphodiesterase GdpP	−9.0		

^1^The Locus tag according to *S. suis* P1/7 genome is provided. ^2^ Putative gene products according to genome annotations. ^3^ Log_2_ fold change decrease found in different sites of infection as compared with inoculum in Tn-Seq. Only fold changes differences lower than 2 are shown. *spec*, spectinomycin-resistance cassette. MTDC, methylene-tetrahydrofolate dehydrogenase/cyclohydrolase; Glu/Gln, Glutamic/Glutamine.

#### Metabolism and transport

Pathogens have evolved a set of metabolic capabilities to facilitate their adaptation to different sites of infection during the infectious process. The Tn-Seq screen identified several genes that participated in diverse metabolic pathways (Table 1). Fig. S4 indicates the localization of genes involved in three major pathways (carbon metabolism, purine metabolism, and amino acid biosynthesis). *S. suis* virulence has previously been found to be associated with specific metabolic activity, in particular with carbohydrate utilization [30,31]. Glycolysis has been proposed as the pivotal route for central carbon metabolism [32], with the exception of *pep* that codes for phosphoenolpyruvate carboxylase (PEP). We detected that PEP was essential for the survival of *S. suis* in blood, CSF, and brain tissues, which is in agreement with previous studies using blood and CSF *ex vivo* models [32]. PEP converts phosphoenolpyruvate to oxaloacetate, an indispensable precursor for *de novo* synthesized amino acids (Asp, Thr) and other biosynthetic metabolites. *S. suis* lacks enzymes for pleiotropic reactions (e.g. phosphoenolpyruvate carboxykinase, malate dehydrogenase, pyruvate carboxylase) to bypass PEP activity, justifying the crucial role of PEP *in vivo*. In addition, *S. suis* lacks an uptake system for Asp and Thr [32]. Together, the dependence of *S. suis* on PEP for survival *in vivo*, in contrast to enriched synthetic medium, may be attributed to the absence of available alternative intermediates in body fluids.

Several components of the pentose phosphate pathway (PPP) were also found to be required for growth of *S. suis in vivo*, comprising genes (*zwF* and SSU0315) that participate in the conversion of glucose-6-phosphate to ribulose-5- phosphate, genes that provide intermediates to the glycolysis pathway (*tkt, deoC)* and downstream reactions (*rpiA, prsA1, deoB*) (Fig. S4A). Many genes were found essential for all infection sites, with few exceptions (*tkt, deoC, deoB*, SSU1315). PPP produces two NADPH molecules per molecule of glucose-6-phosphate through an oxidative pathway branch (*zwf*, SSU1315 and SSU1541). NADPH is a source of electrons for reductases of diverse biosynthetic reactions that repair oxidative damage and regenerate antioxidant species. Deletion of *zwf* in *Salmonella typhimurium*, a bacterium adapted to survival in phagocytic cells, resulted in attenuation of virulence in a mouse infection model but was not attenuated in NADPH phagocyte oxidase knockout mice [33]. As phagocytosis is a relevant immune mechanism for clearance of *S. suis* [34], it is tempting to speculate that the essential role of PPP during *S. suis* infection might be, in part, attributed to the persistence against the antimicrobial activity of phagocytes. In addition, PPP provides precursors for the synthesis of purines and pyrimidines and aromatic amino acids. In concordance, genes involved in these synthesis routes were highly upregulated when bacteria were incubated in blood or CSF in an early study [35]. The correlation with these studies emphasizes the relevance of PPP for *S. suis* virulence possibly linked to the bacterial defense against host immune responses. Moreover, many genes of the pyruvate metabolism pathway (*pdhB, accA, accC, ackA, acoL*) that feed fatty acid biosynthesis (Fig. S4A) appeared to be essential *in vivo* together with genes participating in the fatty acid biosynthesis, for instance, *fabF* and *fabH*. In contrast, genes of the incomplete TCA cycle were found to be dispensable, with exception to *acoL*. Genes that participate in one carbon pool by folate pathway like *fhs*, were also identified in our study and found essential in blood and CSF, while *fhs* was also essential in brain (Table 1 and Fig. S4A). The *fhs* gene codes for folate tetrahydrofolate synthase, which is involved in the synthesis of N^10^-formyltetrahydrofolate and critical for the synthesis of purine nucleotides. Fhs was previously shown to be required for survival of *S. suis* strain SC19 in a mice and porcine model [36]. Accordingly, many components of the purine (n = 13) and pyrimidine (n = 9) metabolism were identified in our *in vivo* Tn-Seq screen (Fig. S4B). Examples included *purA* and *purB* that code for enzymes involved in the conversion of IMP into AMP, among several others, the vast majority of which were required for growth in all infection sites, with few exceptions (*dnaN, polA*, and *tdk*). Because genes of purine biosynthesis were identified, it appears that nucleotide scavenging is essential in the context of the pig infection. It will be relevant to study whether pharmacological perturbation of these enzymes is a feasible approach to treat *S. suis* infections.

*S. suis* strain 10 is auxotrophic for arginine, glutamine/glutamate, histidine, leucine, and tryptophan in CDM medium and this strain has a preference for the acquisition of several amino acids from the environment (glycine, lysine, phenylalanine, lysine, tyrosine, and valine) [31]. Accordingly, mutations in genes encoding enzymes participating in the synthesis of serine from glycerate-3-phosphate (SSU0623, *serC*, SSU1325), alanine (SSU0319), cysteine (*cysE*), glutamine (g*lnA*), proline (*proA, proC*) and homocysteine (*hoM*), a precursor of methionine and threonine appeared detrimental for the infection (Table 1 and Fig. S4 C). Differences were observed within sites of infection, particularly genes coding for enzymes of the synthesis of alanine (SSU0319), conversion of glycine into serine (*acoL*) and synthesis of glutamine by the condensation of exogenous glutamate and NH_3_ (*glnA*), were not essential for the survival of *S. suis* in CSF. Glutamine serves as a nitrogen donor in many biosynthetic processes and can be acquired directly from the environment by using five specific transporters, according to P1/7 genome annotations. The relevance of this enzyme for *S. suis* infection was previously shown in *S. suis* strain SC19 in a murine infection model [37]. Interestingly, our Tn-Seq assays showed that *S. suis* does not require the synthesis of glutamine to survive in CSF and brain tissues as for blood (see *glnA* in Table 1). This is not caused by large differences in the concentration of glutamine between both body fluids [35,38]. Koczuela *et al*. observed higher upregulation of genes encoding for two glutamate/glutamine transporters (SSU0883/0884 and SSU1675/1676) in CSF than in blood [35]. Our Tn-Seq approach identified three conditionally essential glutamine transporters (SSU0883, SSU1675, SSU0447) for blood and brain but not for CSF. These observations point out variation in the acquisition of glutamine at different sites of infection. In *S. pneumoniae*, mutants lacking two glutamine transporters resulted in severe and moderated attenuation of septicemia in a mouse infection model, respectively, and both mutants exhibited different sensitivities to be killed by macrophages [39]. Thus, variations in the acquisition of glutamate/glutamine for *S. suis* could be related to homeostasis or immune evasion at particular sites of infection. Many other genes coding for potential transporters were found to be conditionally essential, some involving uptake of metals. The relevance of these transporters for *S. suis* infection, for instance for magnesium or iron, were already demonstrated in *S. suis* [28,40] or other *Streptococcus* spp. [41,42], some of which are promising targets for antimicrobial therapy or vaccine development [41,43].

Our Tn-Seq approach also identified a large set of genes coding for a total of 22 transporters, many of which located in operons, and involved in the transport of metals (SSU0113-SSU0114, SSU1865-SSU1869), amino acids (SSU0883-SSU0884, SSU1675, SSU1852-1853, SSU0447, SSU501-503), oligopeptides (SSU1662-1664), carbohydrates (SSU1583-SSU1585) and polyamides (SSU0751-SSU0754) (Table 1). The number of essential transporters was higher in isolates from blood (n = 16) than brain (n = 14) and CSF (n = 6). Together, these data reflect the ability of the host to limit access of essential nutrients and the high adaptation of *S. suis* to overcome these limitations.

#### Cell envelope and stress response

The main function assigned to the bacterial cell wall is the preservation of cell integrity through the protection of cells against osmotic stress and host immune defenses such as complement-mediated lysis [44]. We identified essential genes involved in peptidoglycan synthesis and additional structures participating in the formation and regulation of the elongasome and divisome machineries. Several genes coding for enzymes that participated in the biosynthesis of the capsule, cell wall, and membrane were essential for the survival of *S. suis* in pigs (Table 1). This is not surprising as many of those these structures will be first targets of host defenses. Indeed, transposon insertions in almost every gene of the *cps2* gene cluster operon (SSU0516-SSU0526), responsible for the polysaccharide biosynthesis, strongly reduced the capacity of *S. suis* to survive and grow in the three infection sites tested in this study. The capsule is a very well-established virulence factor that contributes to the resistance against phagocytosis and modulation of immune responses and was found to be essential *in vivo* [45–47]. Also, mutants harboring insertions in genes participating directly in peptidoglycan synthesis (*penA, pbp*1a), the elongation (*mreC, mreD*), and cell division apparatus (*gpsB, ftsY, ftsX, ftsE*) were attenuated to growth in pigs (Table 1). Many of those genes are essential for growth under *in vitro* conditions, unless such essentiality is bypassed by suppressor mutations. *mreC, mreD,* and *glsB* are good examples described for *S. pneumoniae* [48–50]. Although these genes may be dispensable *in vitro*, our data evidence that they are essential for *in vivo* survival, but their specific role remains to be investigated. In *S. thermophilus*, a *mreD* mutant exhibited altered cell size and shape and large sensitivity to heat shock [51]. In *Fusobacterium nucleatum*, a deletion mutant in *ftsX* resulted in defective biofilm formation [52] and mutation of *merC* in *Salmonella* affected the expression of virulence factors [53]. Regulators of the cell cycle were also identified to be conditionally essential, such as EzrA, a negative regulator that prevents the formation of aberrant Z ring formation [54,55], an essential structure for the initiation of cell division. Thus, mutations in components of the elongasome and divisome may cause a modification of the peptidoglycan or cytoskeletal architecture that may be of relevance for the resistance of pathogens to stress conditions imposed by the host or for the assembly and coordination of cell surface structures involved on host-pathogen interactions. In addition, mutants with insertion in genes with functions in secretion systems (*ffh, secA, secE*), in many genes encoding hypothetical membrane proteins of unknown function (18 for blood and brain and 10 for CSF), surface anchored proteins and exported proteins (7 for blood and 2 for brain), exhibited a reduced fitness in pigs (Table 1).

The capacity of *S. suis* to survive in the host may also be dependent on its ability to deal with stresses imposed by immune defenses. Certainly, several mutants that participated in stress responses (*grpE, dnaK, tig*) were attenuated for growth at diverse sites of infection (Table 1). The gene encoding the chaperone DnaJ of *S. suis* was shown to contribute to thermotolerance [56], whereas in *S. pneumoniae*, DnaJ was shown to play an important role in inflammatory responses [57]. Trigger factor, another chaperone with peptidyl-prolyl cis-trans isomerization that assists proteins in its proper folding and maturation [58], was shown to contribute to stress tolerance and alter the expression of genes involved in virulence in *S. suis* [59] and other pathogenic streptococci [60]. Besides assisting in stress responses, a role in adhesion was attributed to these proteins [56,60], although adhesion mediated by these cytoplasmic chaperones remains controversial. Even when they lack a signal peptide required for secretion and a cell wall anchor motif, they were partially found at the cell surface [60]. Since such adhesion properties were inferred by the attenuated adhesion capacity of mutants compared to wild-type, it could be possible that these chaperones may affect the assembly or the amount of adhesins present at the surface and therefore their role in adhesion is likely indirect. Our Tn-Seq screen also identified mutants in genes involved in DNA excision and repair (*polA, dnaE, recO*), and genes involved in DNA recombination (*recF*) and replication (*yabA*), which may be essential during host immune responses, e.g. oxidative burst.

#### Transcription regulation

Bacterial signal transduction systems regulate the response to the environmental signal and monitor the condition of the entire cell. A total of 17 genes coding for a large family of regulators (SpxA, CodY, LuxR/S, TetR, Rex, DeoR, LysR, GntR, MarR, CtsR/S, VicC, CcpA, MalR) were detected in our Tn-Seq screens (Table S2 and Table 1). The number of essential regulators varied considerably for blood (*n* = 14), brain (n = 11) and CSF (n = 2), eight of which were uniquely identified in one infection site (five in blood and three in brain) and uniquely one was essential in all sites of infection. Some regulators play a central role in metabolism and virulence. Examples include CcpA, CodY, and LysR. CcpA controls the expression of conserved genes involved in the carbohydrate metabolism [61] and virulence [62,63]. In *S. suis*, especially in serotype 2, *ccpA* mutant derivatives exhibited a reduced expression of several virulence genes compared to the parent strain such as *sly, sao, eno,* or genes participating in capsule biosynthesis [31,64], and were found to be attenuated in virulence in mice infection models [64]. CodY controls several metabolic routes like biosynthesis of branched-chain amino acids, carbon metabolism, or uptake of different components (peptides, sugars, metals) [65], particularly in *Streptococcus* spp [66,67]., and surface structures including capsule thickness and sialic acid synthesis, in agreement with the essential role of genes involved in capsule biosynthesis. An *S. suis* mutant lacking *codY* showed reduced adherence of *S. suis* to HEp-2, enhanced sensitivity to phagocytosis, and attenuated virulence in a BALB/c mice infection model [68]. The LysR-type transcriptional regulators mediate the metabolism and uptake of amino acids, but their role in *S. suis* was not studied. *S. suis* SSU0869 encodes for a LysR regulator with homologies to regulators of the methionine biosynthesis and transport. In agreement with its putative essential role in *S. suis* infection is the identification of genes involved in the uptake of methionine. Other identified regulators are associated with stress responses, for example, CtsR, Rex, and SpxA. They regulate stress response through the expression of stress genes encoding for Clp ATPases and ClpP protease [69,70] and heat shock responses, both of considerable relevance for moderate survival and virulence of pathogens [71]. In several *Streptococcus sp*, SpxA regulators are involved in growth under oxidative stresses acting on several oxidative stress genes (*dpr, nox, sodA* and *tpx*) [72,73], confirming the identification of *nox* in our Tn-Seq screen. Based on these results, we conclude that *S. suis* uses a balanced network of regulators to control metabolic adaptation and stress responses during infection. In our previous work, we showed that metabolic adaptation of *S. suis* to the different sites of infection is causing large changes in gene expression [21]. In this study, it is shown that these metabolic changes are not facultative, but essential for *S. suis* in order to survive in the harsh conditions of the host. Future studies to investigate the exact regulation of gene expression to adapt to the different niches in the host will help to complete the essential regulatory network of *S. suis* to sense and survive in the host environment.

#### Virulence factors

The high abundance of essential genes related to metabolism, transport, transcription, and regulation contrasted with a minor pool of genes encoding known proteins and structures considered as virulence factors for the infection. Well-known virulence factors of *S. suis* like Muramidase-released protein, Suilysin, extracellular factor, fibrinogen binding proteins, or Hyaluronate lyase [34] were missing in our list of conditionally essential genes. This could be caused by the fact that most host barriers were bypassed due to direct inoculation into the subarachnoid space. Thus, those virulence factors playing a role in breaching the host barriers by promoting adhesion or invasion were not required, since the *in vivo* selection was done by direct inoculation into the CNS. However, one would expect to find mutations in some factors like Enolase or Suilysin, which play a role preventing phagocytosis [74], a relevant immune mechanism to fight against *S. suis* infection [2]. Possibly, the redundancy of factors involved in similar functions resulted in the dispensability of these factors.

### *Role of selected essential genes under* in vivo *mimicking conditions*

**Figure 3.**
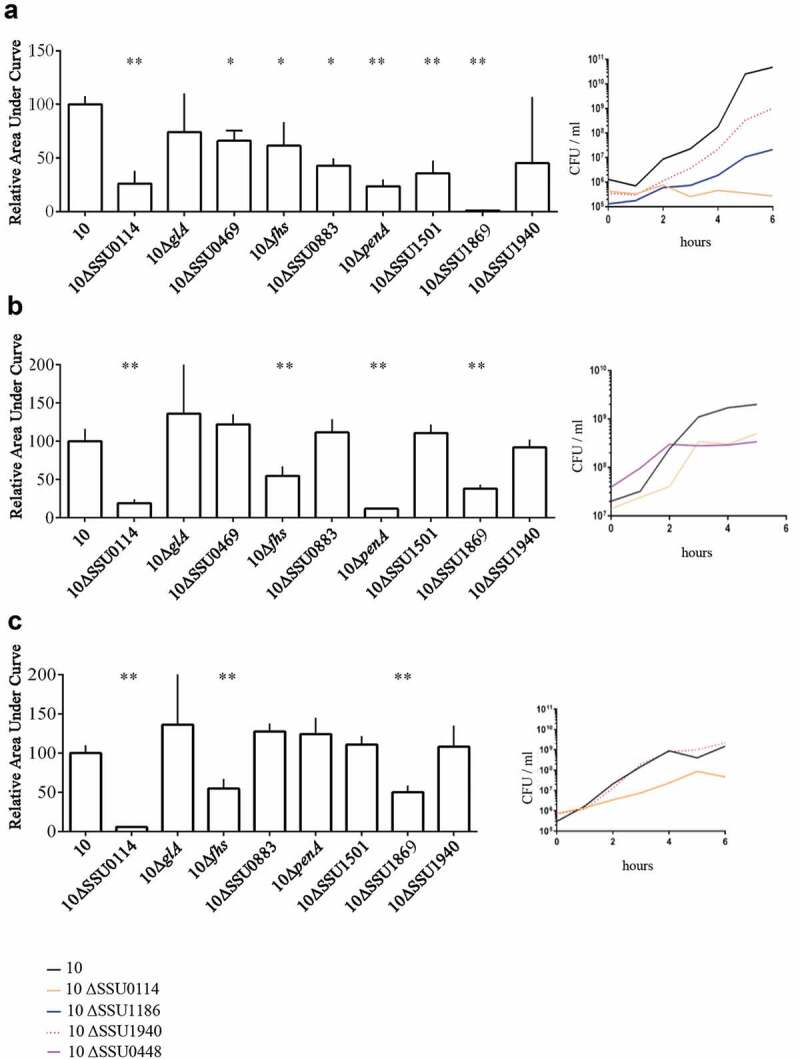
Survival of *S. suis* strain 10 and derivative mutants in *ex vivo* (a) porcine blood, (b) porcine serum, and (c) porcine CSF. Bacteria were cultured in THB medium, diluted, and then incubated in the different body fluids. The number of bacteria was determined by CFU counting at time 0 and every 2 h over a time course of 6 h. Data are expressed as the total area under the curve obtained in three independent experiments and relative to that of the wild type (left) representative growth curves for selected strains are shown (right). One or two asterisks indicate statistically significant differences compared to the wild type at P < 0.05 and P < 0.001, respectively.

**Figure 4.**
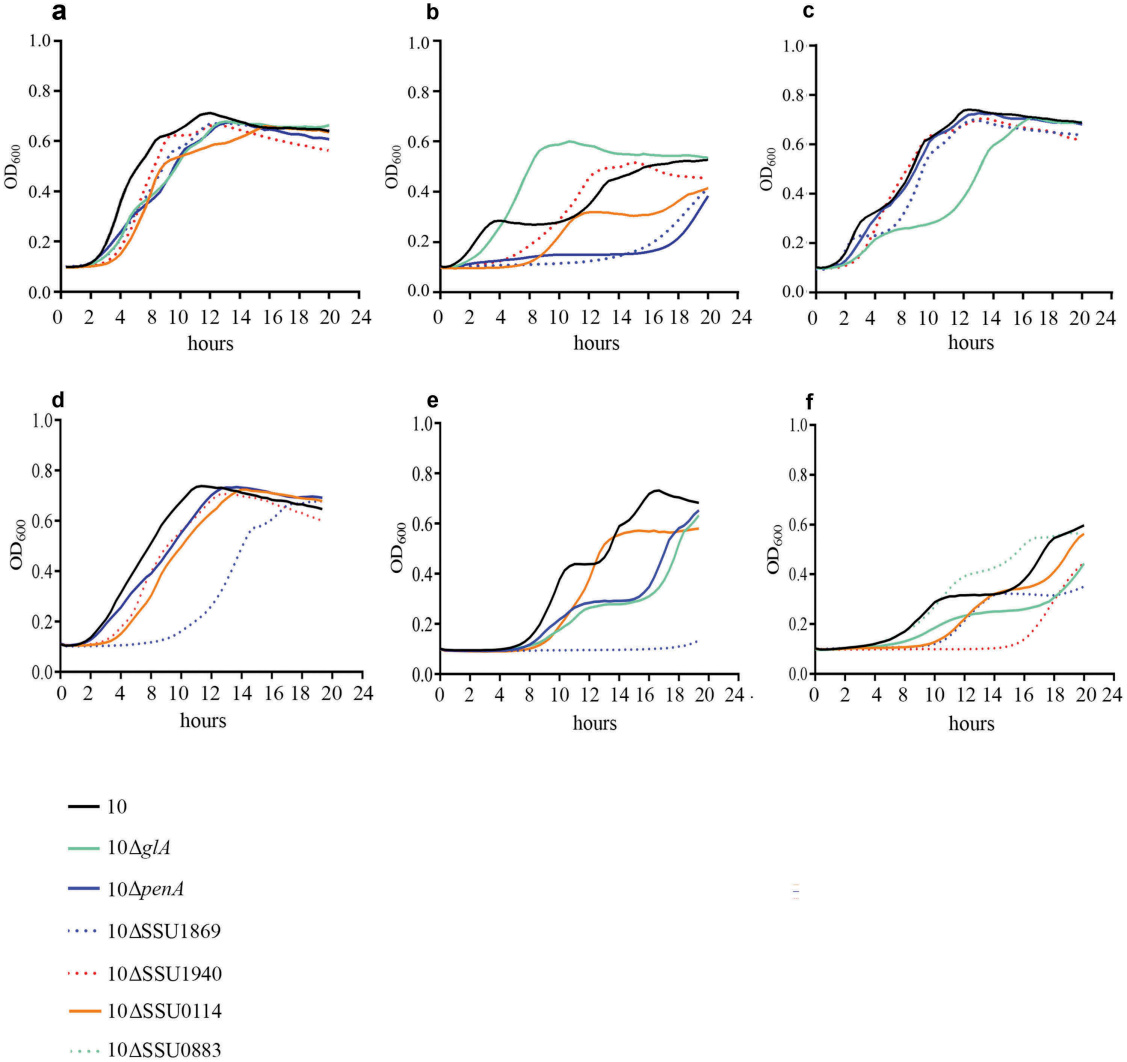
Growth curve of *S. suis* strain 10 and mutant derivatives under different environmental conditions. Bacteria were cultured in THB medium (a, d, e, f) at 37̊C, (b) at 42̊C and (c) at 34̊C in presence of (d) 5 mM NaF, (e) 2 mM H_2_O_2_ and (f) 1 mM of Bipyridyl, and the optical density at 600 nm (OD_600_) was measured every 20 minutes over a time course of 18 hours.

To confirm our essential gene set and to gain insights into their potential role during infection, we selected a subset of genes. Our criteria for selection were those genes coding for proteins 1) belonging to different functional categories, 2) differently located at the bacterial cell, and 3) diversely required at sites of infection according to our Tn-Seq results (Table 1). Thus, we selected those genes that code for an ABC transporter permease (a.k.a. TroC, SSU0114), a putative glycerol-uptake facilitator aquaporin (a.k.a. GlpF, *glA*), a maf-like protein of unknown function (SSU0469), the Formate-tetrahydrofolate ligase (*fhs)*, a glutamine ABC transporter (a.k.a. GlnQ, SSU0883), the penicillin-binding protein 2b (a.k.a. PBP2B, *penA*), a CsbD-like protein (SSU1501), a metal-binding lipoprotein (a.k.a. TroA, SSU1869) and the c-di-AMP phosphodiesterase (a.k.a. GdpP, SSU1940). We generated directional deletions of each gene by replacing the entire encoding sequence with a spectinomycin-resistance cassette and assessed bacterial survival in different *in vivo* mimicking conditions. In porcine body fluids, *S. suis* encounters a diversity of nutritional limitations, stresses, and immune defenses. Thus, bacteria were incubated in porcine whole blood, porcine serum, and CSF to emulate *in vivo* conditions, and bacterial survival was monitored at various time points through CFU determination. With exception to mutants 10∆*glA* and 10∆SSU1940, which showed a large variation within assays, all mutants exhibited significant attenuation to growth in porcine blood as compared to the wild-type strain (Figure 3(a)). This confirmed that the selected genes are indeed crucial for the survival of the pathogen during septicemia. Mutant strains were then assessed for their growth in porcine sera using the same incubation conditions. *S. suis* mutants 10∆SSU0114, 10∆*fhs*, 10∆*penA,* and 10∆SSU1869 were attenuated in growth in porcine sera (Figure 3(b)), which, with exception to *penA*, is consistent with their assigned function in metabolism and transport. The remaining mutants grew as efficiently as wildtype strain 10. A set of mutants was incubated in porcine CSF. *S. suis* strains 10∆SSU0114, 10∆*fhS,* and 10∆SSU1869 were inhibited in growth in porcine CSF (Figure 3(c)). Thus, the ability of the bacteria to survive in host fluids was reduced in the subset of selected mutants compared to the parent strain, confirming our *in vivo* Tn-Seq screen. Strain 10 and mutant derivatives were also analyzed for their growth in rich THB medium at 37°C under the same conditions used for the incubation with host fluids. While mutants 10∆SSU0114, 10∆*glA*, 10∆*penA*, 10∆SSU1869, and 10∆SSU1940 exhibited a slight retardation of its logarithmic phase as compared to the parent, the growth for the remaining mutants was unaltered (Figure 4(a) and data not shown). Therefore, we conclude that the phenotypes observed in host fluids were not attributable to growth defects in THB, even for those mutants that did show slight retardation. We then tested bacteria growth at 42°C in THB to understand whether fever was also responsible for the restricted growth of some Tn-Seq mutants during in vivo infection. Heat shock alters membrane permeability resulting in the impairment of proton motive force. Additionally, it causes protein unfolding leading to the formation of protein aggregates that can lead to cell death. To cope with high temperature, bacteria use a heat shock response constituted by sensors, transcriptional regulators, and chaperones that mediate protein folding and degradation of denatured proteins. *S. suis* strain 10 exhibited an alteration of the growth at 42°C as compared to 37°C (compare Figure 4(a,b)); in particular, the growth was depressed between 4 and 8 h after which was restored. These results suggest that strain 10 requires an adaptation to a high temperature to allow optimal growth, possibly as a result of the activation of the heat shock response. Mutants 10ΔSSU1869, 10ΔSSU0114, 10Δ*penA*, and 10ΔSSU1940 exhibited an attenuated growth rate as compared to the parent (Figure 4(b)). The sensitivity of the Δ*penA* mutant to high temperature could directly be caused by an enhanced sensitivity of an unorganized cell wall. In contrast, mutations in components of metal transporters (SSU1869, SSU0114) could be linked to the requirements of metals for activating and stimulating enzymes involved in thermotolerance. For example, SSU1869 encodes a TroA protein, part of an ABC transporter involved in Manganese uptake in *S. suis* [28], while SSU0114 is part of another ABC transporter which substrate has not been elucidated. The heat shock chaperone GroEL requires divalent cations such manganese for ATPase activity and stabilization likely imported by the cited transporters [75]. Other chaperones such as DnaJ, which possess a demonstrated function in thermotolerance of *S. suis* serotype 2 [56], needs Zinc for the recognition of substrates [76]. In contrast to the cited mutants, mutant 10∆*glA* lacked a depressed logarithmic phase as observed for the wild type (Figure 4(b)). To identify whether these proteins encoded by mutated genes were indeed thermic shock proteins, we tested growth at 34ºC. *S. suis* strain 10 exhibited a similar growth at 34°C as at 37°C (compare Figure 4(a,c)), but in contrast to the profiles observed at 42°C, the mutants 10ΔSSU1869, 10ΔSSU0114, and 10Δ*penA* exhibited a similar growth as the parent (Figure 4(c)). This suggests that these genes participate in the thermotolerance of *S. suis* to fever. In contrast, the mutant 10∆*glA* exhibited an elongated logarithmic phase at 34°C (Figure 4(c)).

To understand the role of these genes in nutritional immunity and additional stress conditions, we incubated wild type and mutant derivatives in THB medium with a set of different growth-inhibitor agents: 0.5 mM 2,2-Bipyridyl (Bipyridyl), 10 mM NaF, and 3 mM H_2_O_2_. Bipyridyl is a chelator of transition metals that is used to mimic growth conditions *in vivo* where host metals are not available due to metal-binding host proteins. NaF is an inhibitor of intracellular enzymes such as membrane-bound ATPase [77,78], among others, generating cell stress conditions. H_2_O_2_ penetrates bacterial membrane and interacts with ferrous iron-producing reactive hydroxyl radicals that cause irreversible cellular damage. The addition of H_2_O_2_ may mimic *in vivo* conditions where free radicals are formed during phagocytosis. Supplementation of the growth culture medium with NaF minimally affected the growth of *S. suis* strain 10 (compare Figure 4(a,d)) while supplementation with H_2_O_2_ and bipyridyl retarded its logarithmic phase (compare Figure 4(a,e,f)). NaF minimally affected the growth of mutant derivatives 10∆SSU0114, 10∆*penA*, and 10∆SSU1940, which exhibited a slight retardation of its logarithmic phase, and severely the growth of the mutant 10∆SSU1869 (Figure 4(d)). These analyses evidence the requirements of at least Mn^+2^ for the resistance of *S. suis* to NaF. The reasons for the sensitivity of these mutants are unknown and it might be related to the reduced attention that antimicrobial activity of fluoride to *S. suis* has received. In presence of H_2_O_2_, mutant strains 10∆*glA* and 10∆*penA* exhibited a substantial growth defect as compared to the wild type (Figure 4(e)), while the mutant 10∆SSU1869 did not grow (Figure 4(e)). The results obtained with 10∆SSU1869 are in full agreement with those previously obtained in a virulent *S. suis* isolate of serotype 9 [28], confirming that TroA indeed enhances tolerance to oxygen radicals. One possible explanation is that the deletion of TroA reduces intracellular manganese levels and subsequently the activity of manganese-dependent superoxide dismutase, which protects the cell from superoxide. Besides that, addition of Bipyridyl to the culture medium drastically retarded the logarithmic phase of the derivative mutants 10Δ*glA*, 10ΔSSU0114, 10ΔSSU1869, and 10∆SSU1940 and enhanced that of the mutant 10∆SSU0883 as compared to the wildtype (Figure 4(f)). Results suggest that ions sequestered by bipyridyl could be substrates for encoded transporters and that, likely, some of them are required for GdpP activity. Overall, our assays mimicking *in vivo* conditions evidenced that many of the selected genes are involved, directly or indirectly, in resistance of *S. suis* to stress conditions generated during the infection.

### Co-infection experiments in animals

**Figure 5.**
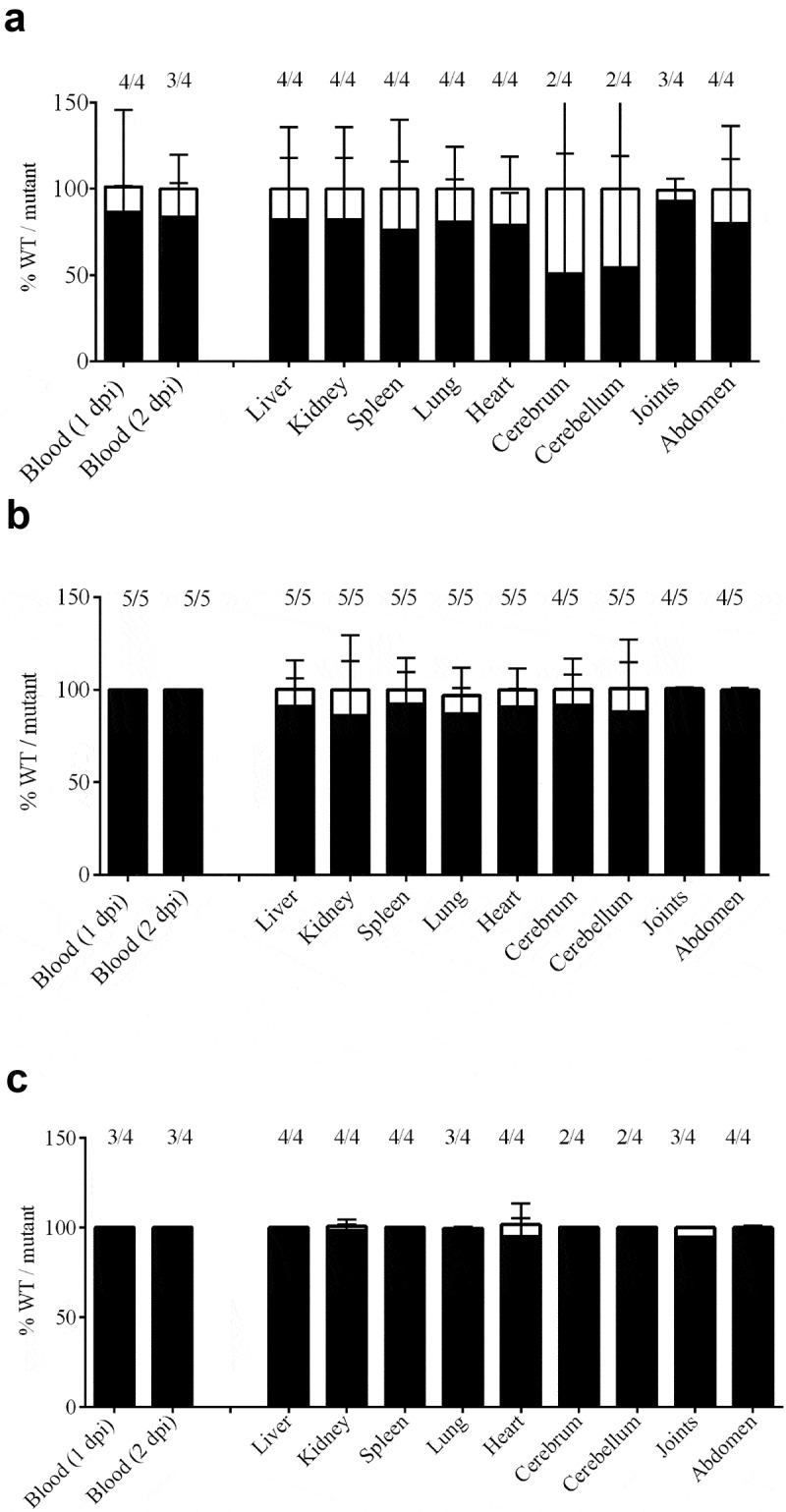
Bacterial load in blood and organs after intravenous infection of pigs with strain 10 and mutant derivatives (a) 10∆*penA*, (b) 10∆SSU1869, and (c) 10∆SSU1940. Cells of strain 10 and mutant derivatives carrying a spectinomycin-resistance marker, were mixed 1:1 and the resulting suspensions were used to infect animals. Bacterial loads of the wildtype and mutants in blood, one- and two-day post-infection (dpi), in internal organs and brain, four dpi, were determined by plating on solid media containing or not spectinomycin and counting colony-forming units after overnight incubation. The ratio wild type/mutant is shown and the number of animals that resulted positive for each strain is depicted at the top. The ratio wildtype/mutant in blood remained similar to the three dpi.

To confirm the role of selected genes in the infection process *in vivo* we performed co-infection experiments. We hypothesized that mutants lacking conditionally essential genes for survival during infection *in vivo* will be less fit as compared to the wild type. We selected three mutants that showed pronounced phenotypes in several *in vitro* analysis: 10∆SSU1869, 10∆SSU1940, and 10∆*penA* (Figures 3 and 4). Hence, bacterial suspensions of *S. suis* wild type and selected mutant derivatives were mixed at ratio 1:1 and used to infect piglets. All piglets developed fever within 24 hpi. White blood cell counts in blood increased significantly on day 1 post-infection; all piglets reached humane endpoints due to *S. suis* specific symptoms within 3 days post-infection. To determine the fitness of the mutant isolates, compared to the wild strain 10, the ratio of the wild-type strain 10 and the mutants were determined by selective plating. The ratio between wild type and mutant in blood was determined on a daily base. Results are shown in Figure 5. Strain S10Δ*penA* was outgrown by the wild-type strain 10 in blood, but not completely disappeared. A putative role in virulence was early described for PBP2. A mutant in *penA* showed reduced adherence to epithelial cells in *S. uberis* and was identified using a Tn-Seq strategy [79]. However, as discussed earlier, a putative role in adherence to epithelial cells cannot be hypothesized to *S. suis* PBP2 from our work, as we directly inoculated bacteria into the blood. A plausible explanation is that the deletion of *the penA* affected the function and/or exposition of virulence determinants attached to the cell wall. Besides that, a putative role in virulence was previously proposed for TroA, as the corresponding mutant in an *S. suis* serotype 9 strain was attenuated in a murine infection model [28]. Strain 10ΔSSU1869 could not be re-isolated from blood from day 1 post-infection onwards, and could hardly be re-isolated from organs, confirming a relevant role of this uptake system *in vivo* in a pig infection model. Similarly, strain S10ΔSSU1940 could not be re-isolated from blood and could only be re-isolated from organs of one piglet during necropsy resulting even more attenuated than strain 10 ΔSSU1869. This is in accordance with data from *S. pneumoniae*, where two DHH proteins were identified using Tn-Seq and were shown to be involved in virulence *in vivo* [80]. One of these proteins, PapP (SP1298), was found to be required for the maintenance of membrane lipid homeostasis explaining the attenuation of virulence [81].

To summarize, in this work we identified genes of *S. suis* with a lower fitness to survive *in vivo* during an infection in pigs using a Tn-Seq strategy. This was done using a novel porcine infection model to induce meningitis by direct intrathecal inoculation of the subarachnoid space. By this route, the bacterium bypasses several host barriers, including, for example, mucosal immunity. This prevents bottlenecks during the infection, an important limitation of Tn-Seq studies i*n vivo*. Although other infection models can nicely emulate the whole course of the infection, e.g. intranasal infection models, and putatively could identify genes required for colonization and invasion of the host, these models have a high risk of re-isolating too low number of bacteria and thus they are not useful in combination with Tn-Seq approaches. Moreover, the current intrathecal meningitis model in pigs can be applied broadly than for *S. suis* infection. Pigs are a very good model for testing human pathogens due to the high similarity in physiology and anatomy between pigs and humans. Thus, very likely, this model could be used for human meningitis studies, for instance into pneumococcal, cryptococcal, or coccidioidal meningitis. Host immune responses can be studied using this model. This model can be also used to test novel dedicated intrathecal treatments that require preclinical studies to determine toxicity and effectivity in animal models. Together, our porcine model is broadly applicable in the veterinary and human field of infectious diseases.

Here, 361 genes of *S. suis* serotype 2 were identified as conditionally essential for meningitis. Growth of directional mutants under *in vivo* mimicking conditions confirmed the growth attenuation. Decreased virulence of three of the mutants was demonstrated under experimental conditions in pigs, proving the role of these mutants in the survival of *S. suis in vivo*. Our results highlight the power of the Tn-Seq strategy for evaluation of the genetic requirements for bacterial survival in the host; therefore, it will be the foundation of future studies focused on streptococcal meningitis. Moreover, several of the identified conditionally essential genes are worth additional research to screen their potential as vaccine candidates. Both live-attenuated vaccines, subunit vaccines and novel treatment therapies can be based on our data.

## Supplementary Material

Supplemental Material
