# Supplementary material for: Identification of conditionally essential genes for Streptococcus suis infection in pigs
Source: Virulence. 2020 May 18;11(1):446–64. doi: 10.1080/21505594.2020.1764173 (PMC7239030; doi:10.1080/21505594.2020.1764173)
Supplement: Supplemental Material [file KVIR_A_1764173_SM3586.zip › Figure_S1.pdf]

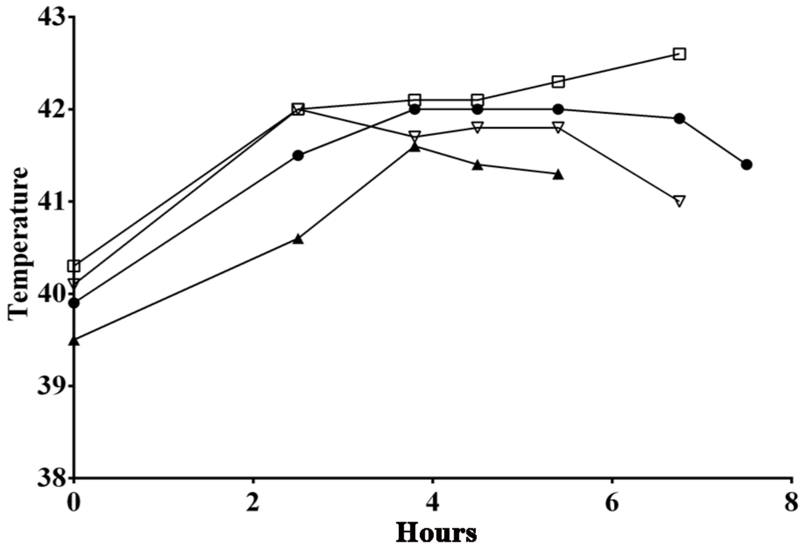

Figure S1. Clinical features of pigs infected intravenously and epidurally with strain 10. The rectal temperature was evaluated at time 0 and each hour after infection over a time course of 8 hours. Each symbol represents one individual animal.
