# Supplementary material for: Identification of conditionally essential genes for Streptococcus suis infection in pigs
Source: Virulence. 2020 May 18;11(1):446–64. doi: 10.1080/21505594.2020.1764173 (PMC7239030; doi:10.1080/21505594.2020.1764173)
Supplement: Supplemental Material [file KVIR_A_1764173_SM3586.zip › Figure_S2.pdf]

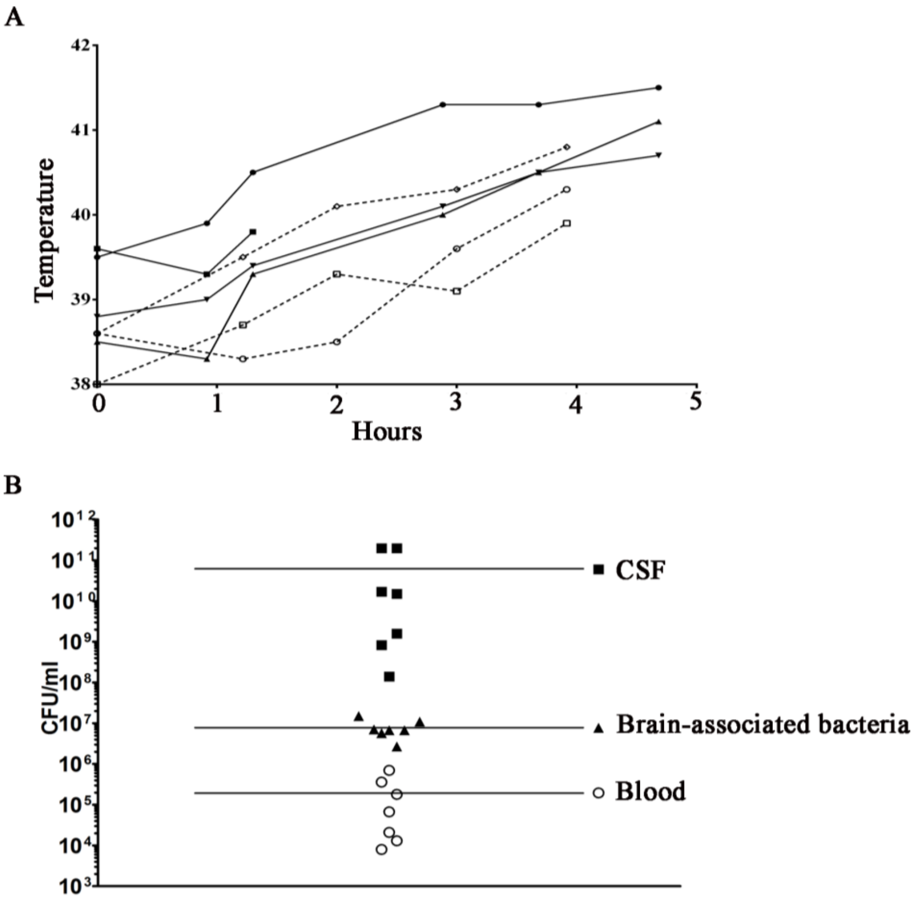

Figure S2. Clinical symptoms and bacteriology analysis in 8 piglets after experimental infection with *S. suis* strain 10 Tn-Seq mutants. (A) The rectal temperature was evaluated at time 0 and each hour after infection over a time course of 5 hours. (**B)** Bacterial loads in blood, CSF and brain after 5 hours of infection as determined by CFU determination. The horizontal bar represents the average for each group. Each symbol represents one individual animal.
