# Supplementary material for: Identification of conditionally essential genes for Streptococcus suis infection in pigs
Source: Virulence. 2020 May 18;11(1):446–64. doi: 10.1080/21505594.2020.1764173 (PMC7239030; doi:10.1080/21505594.2020.1764173)
Supplement: Supplemental Material [file KVIR_A_1764173_SM3586.zip › Figure_S3.pdf]

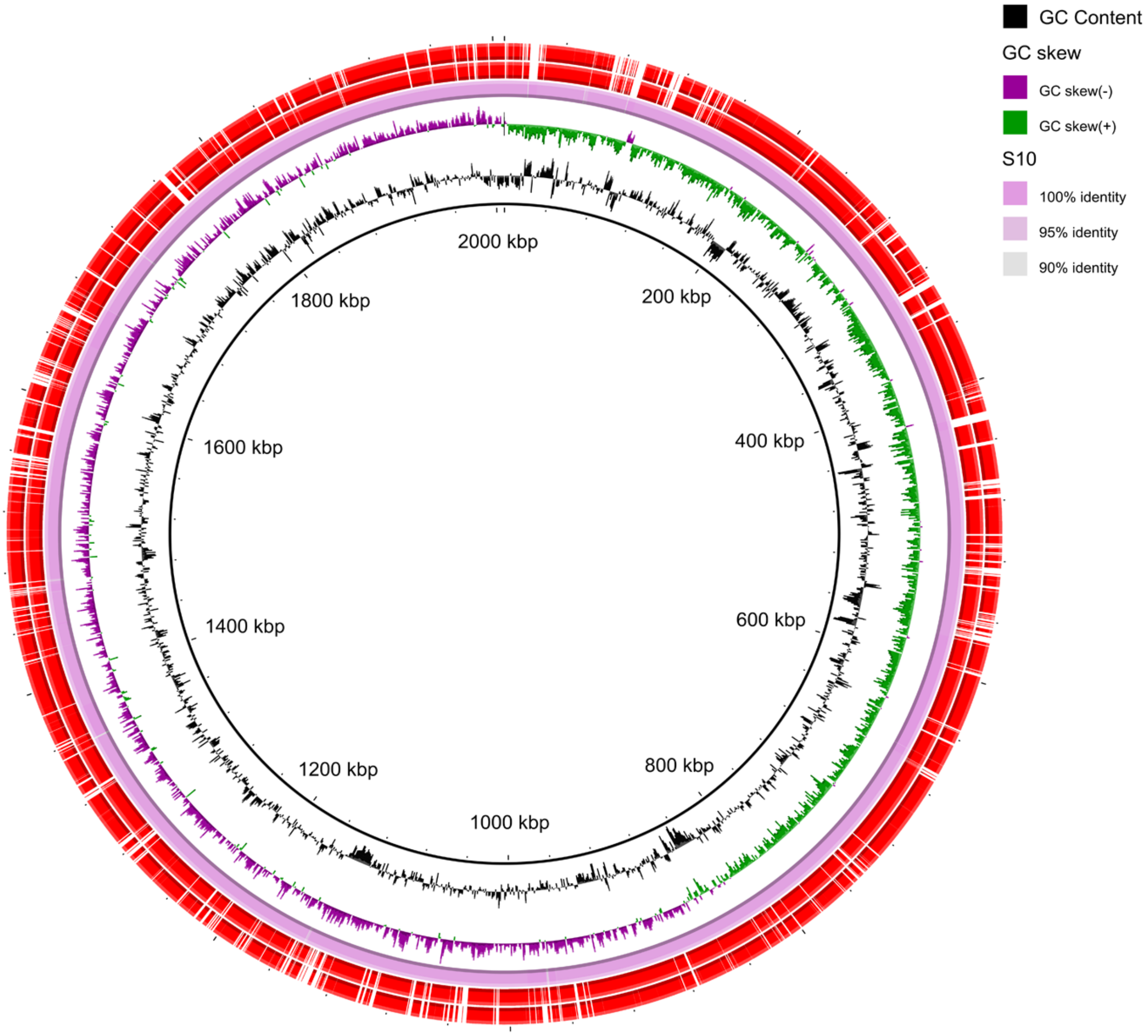

Figure S3. Map of Tn-Seq mutations in *S. suis* libraries used in this study. The inner ring represents the chromosome of public available P1/7 genome. Also depicted are the GC Skew and GC content. The pink ring shows a genome comparison of *S. suis* strain 10 with P1/7 genome. The outermost red ring depicts the position of Tn-Seq mutations detected after pig infection (output) and the second most outer ring depicts the Tn-Seq mutations in the inoculum (input).
