# Supplementary material for: Identification of conditionally essential genes for Streptococcus suis infection in pigs
Source: Virulence. 2020 May 18;11(1):446–64. doi: 10.1080/21505594.2020.1764173 (PMC7239030; doi:10.1080/21505594.2020.1764173)
Supplement: Supplemental Material [file KVIR_A_1764173_SM3586.zip › Figure_S4.pdf]

A) Carbon metabolism

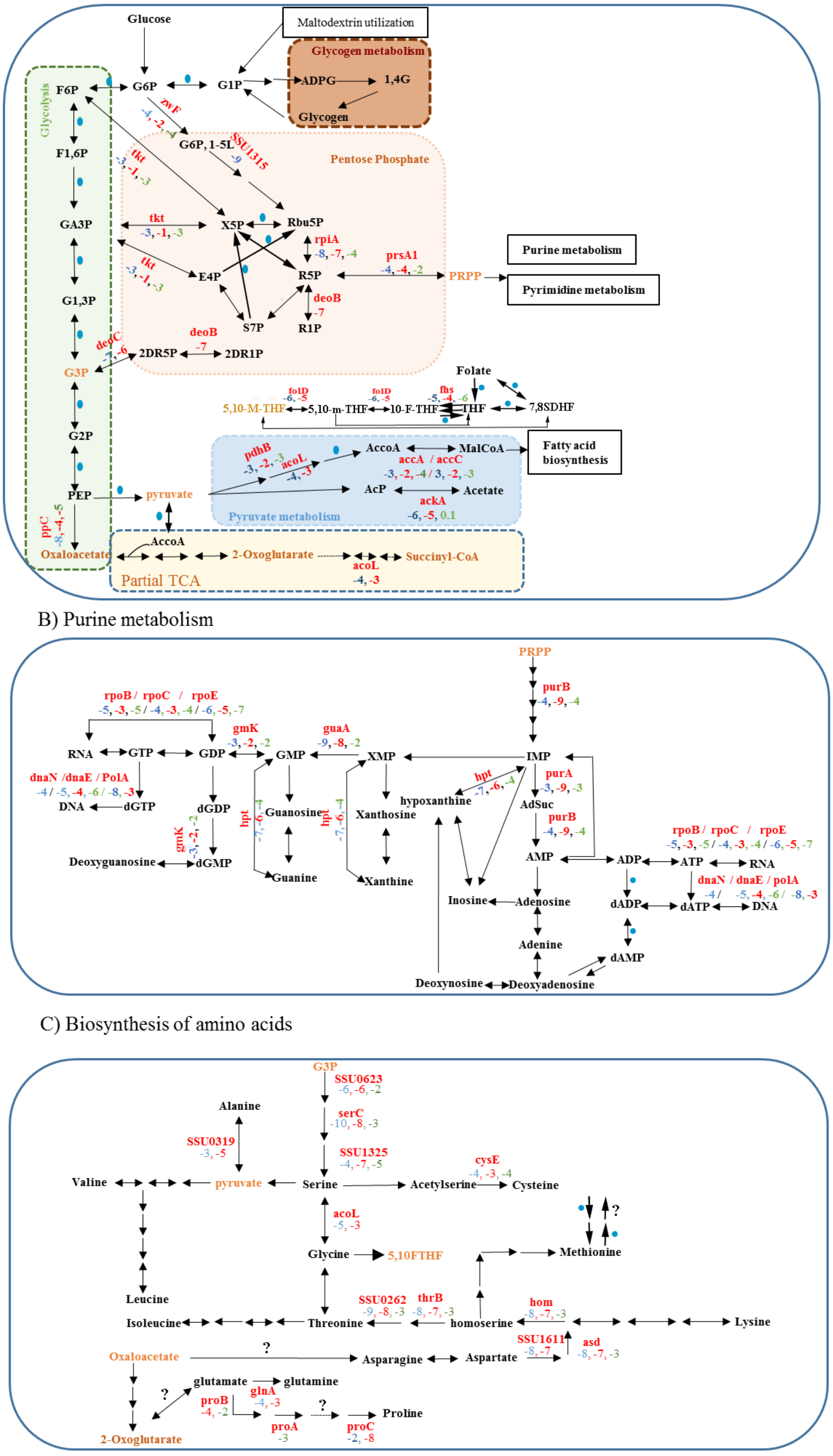

Fig S4. Essential genes for *S. suis* infection participating in three metabolic pathways. The respective genes or locus tags, according with P1/7 genome, are indicated in red. Genes were considered essential based on a $\log_2$ fold change decrease of at least two as compared with input inoculum and an adjusted *P* value lower than 0.05. The fold change decrease for each gene after analysis of transposon mutant library recovered from blood (blue), CSF (red) and brain (green) is depicted. Pathways were predicted based on genome information and KEGG database survey. Blue dots indicate essential genes in the inoculum.

Abreviations:

G6P, Glucose 6-Phosphate; F6P, Fructose 6-Phosphate. F1,6P Fructose 1,6-Phosphate; GA3P, glyceraldehyde-3P; G1,3P, glycerate 1,3 Phosphate; G3P, Glycerate-3 Phosphate; G2P, Glycerate-2 Phosphate; PEP, Phosphoenolpyruvate; CoA, Coenzyme A: AccoA, Acetyl Coenzyme A; Malonyl CoA, Malonyl Coenzyme A; AcP, Acetyl phosphate; THF, Tetrahydrofolate; 10-F-THF, 10 formyl-THF;
5,10-m-THF, 5,10-Methenyl-THF; 5,10-M-THF, 5,10-Methylene-THF; 7,8-Dihydrofolate;5,10FTHF, 5,10-Methylenetetrahydrofolate. G6P, 1,5L D-Glucono-1,5-lactone 6-phosphate; Rbu5P, D-Ribulose 5-phosphate; R5P, D-ribose 5- phosphate; X5P, D-Xylulose 5-phosphate. S7P, D-Sedoheptulose 7-phosphate; E4P, D-Erythrose 4-phosphate; PRPP, 5-Phospho-alpha-D- ribose 1-diphosphate. R1P, D-Ribose 1-phosphate. 2DR5P, 2-Deoxy-D-ribose 5-phosphate; 2DR1P, 2-Deoxy-alpha-D-ribose 1-phosphate; ADPG, ADP-glucose; 1,4G, 1,4-alpha-D-Glucan; dGDP, 2'-Deoxyguanosine 5'-diphosphate; dGTP; 2'-Deoxyguanosine 5'-triphosphate; dGMP; 2'-Deoxyguanosine 5'-monophosphate; XMP, (9-D-Ribosylxanthine)-5'-phosphate; IMP, Inosine 5'-monophosphate; ADSuc, Adenylosuccinate; dADP, 2'-Deoxyadenosine 5'-diphosphate; dAMP, 2'-Deoxyadenosine 5'-phosphate; dADP; 2'-Deoxyadenosine 5'- diphosphate; Zwf, glucose-6-phosphate 1-dehydrogenase ; tkt, putative transketolase; rpiA; ribose 5-phosphate isomerase A; deoB, phosphopentomutase deoC; deoxyribose-phosphate aldolase; PrsA1; ribose-phosphate pyrophosphokinase 1; ppc; putative phosphoenolpyruvate carboxylase; fhs; formate--tetrahydrofolate ligase; FolD bifunctional protein [includes: methylenetetrahydrofolate dehydrogenase/methenyltetrahydrofolate cyclohydrolase]; pdhB; putative pyruvate dehydrogenase E1 component, beta subunit; acoL; dihydrolipoamide dehydrogenase; accA, acetyl-coenzyme A carboxylase carboxyl transferase subunit alpha; accC, biotin carboxylase subunit of acetyl-CoA carboxylase; ackA, acetate kinase; dnaN, DNA polymerase III, beta chain; dnaE, DNA polymerase III alpha subunit, polA; DNA polymerase I. rpoB; DNA-directed RNA polymerase beta chain; rpoC, DNA-directed RNA polymerase beta' chain; rpoE, putative DNA-directed RNA polymerase, delta subunit; gmk, guanylate kinase; guaA; GMP synthase [glutamine-hydrolyzing]; hpt, hypoxanthine-guanine phosphoribosyltransferase; purA, adenylosuccinate synthetase; purB, adenylosuccinate lyase; serC, putative phosphoserine aminotransferase; acoL; dihydrolipoamide dehydrogenase; cysE, serine acetyltransferase; thrB; homoserine kinase. Hom, homoserine dehydrogenase; asd, aspartate-semialdehyde dehydrogenase; glnA, putative glutamine synthetase; proB, glutamate 5-kinase; proC, putative pyrroline-5-carboxylate reductase; proA; gamma-glutamyl phosphate reductase.
