# Supplementary material for: Identification of conditionally essential genes for Streptococcus suis infection in pigs
Source: Virulence. 2020 May 18;11(1):446–64. doi: 10.1080/21505594.2020.1764173 (PMC7239030; doi:10.1080/21505594.2020.1764173)
Supplement: Supplemental Material [file KVIR_A_1764173_SM3586.zip › Table_S1.pdf]

Table S1. Primers used in this study.

Target Gene[a]	Putative function	Primer	Sequence (5'- 3')
SSU0144	Permease of a metal ABC- transporter	Fssu0114-L1	gtggggaatttgtgactt
		Rssu0114-L2	acgaacgaaaatcgacctgcaaactttgccgcctgag
		Fssu0114-R2	ttagaaaacaataaacccttgcataatggttatggggttgctgacttc
		Rssu0114-R1	agcatctttcttttctgggtattt
glA	Aquaporin	Fssu0249-L1	caccgacaaagttgaaaaggatac
		Rssu0249-L2	acgaacgaaaatcgacctgc ataataaggagtgcagtcgctaaa
		Fssu0249-R2	ttagaaaacaataaacccttgcat catcaattgccgctgtt
		Rssu0249-R1	cttgtccaaatcgcccacctc
SSU0469	unknown	Fssu0469-L1	agcaactcttatccaaccaacct
		Rssu0469-L2	acgaacgaaaatcgacctgcataatctcctgaaaaacctgacca
		Fssu0469-R2	ttagaaaacaataaacccttgcatactcccacccccgcacag
		Rssu0469-R1	tacccagtcagtcgataataagc
fhs	Formate-tetrahydrofolate ligase	Fssu0648-L1	atcagcgccatttcgttca
		Rssu0648-L2	acgaacgaaaatcgacctgctgtgcaatgtcaatatctgttttc tta
		Fssu0648-R2	gaaaacaataaacccttgcatgtggcagcagacgggacag
		Rssu0648-R1	tggggtgcagctatttcaggt
SSU0883	Permease of a Glu/Gln ABC-Transporter	Fssu0883-L1	gtcacgggaagctgggaaaatct
		Rssu0883-L2	acgaacgaaaatcgacctgctaaactcgaatggggcgtaagc
		Fssu0883-R2	ttagaaaacaataaacccttgcat aactcttccaagcgggtaa
		Rssu0883-R1	acattgatatttcggcgtttct
penA	Penicillin-binding protein 2b	Fssu1186-L1	ccttagtgggggcgaatgg
		Rssu1186-L2	acgaacgaaaatcgacctgcaaaatcacgatagaaaataaaata
		Fssu1186-R2	ttagaaaacaataaacccttgcatgcggtagcagttgtcct
		Rssu1186-R1	tccccggtctggctaatgaaata
SSU1501	CsbD-like protein	Fssu1501-L1	ttgtcatggactcatgtgac
		Rssu1501-L2	acgaacgaaaatcgacctgcttgcagatccagatagctg
		Fssu1501-R2	ttagaaaacaataaacccttgcataagagctagtcgaagatgc
		Rssu1501-R1	cagagagggcacagatgagg
SSU1869	Permease of an ABC transporter		
		OZnu F	aaatagtcggaatgacagcactgg
		OZnu R	ctatgccgtaggtcgcctgataa
SSU1940	DHH family protein	Fssu1940-L1	cttgaccagtcgggcagaata
		Rssu1940-L2	acgaacgaaaatcgacctgcacagcgcaatcaacaccaatag
		Fssu1940-R2	ttagaaaacaataaacccttgcataggcggtattcgttgta
		Rssu1940-R1	attgcctcatttctatcag
Spectinomycin		Spec-F	gcaggtcgattttcgttcgt
		Spec-R	atgcaagggtttattgttttctaa

[a] with exception of spectinomycin, gene name or gene locus based on genome sequences of P1/7 are provided.
